# HLA-DQB1 and HLA-DRB1 Variants Confer Susceptibility to Latent Autoimmune Diabetes in Adults: Relative Predispositional Effects among Allele Groups

**DOI:** 10.3390/genes10090710

**Published:** 2019-09-13

**Authors:** Minting Zhang, Shuhuang Lin, Xiaoling Yuan, Ziqi Lin, Zunnan Huang

**Affiliations:** 1 Key Laboratory for Medical Molecular Diagnostics of Guangdong Province, Dongguan Scientific Research Center, Guangdong Medical University, Dongguan 523808, China; hjzxzmt@163.com (M.Z.); shuhuang_lin@yahoo.com (S.L.); 2 The Second School of Clinical Medicine, Guangdong Medical University, Dongguan 523808, China; 3 School of Public Health, Guangdong Medical University, Dongguan 523808, China; 15622906467@163.com; 4 School of Pharmacy, Guangdong Medical University, Dongguan 523808, China; 13192477423@163.com; 5 Institute of Marine Biomedical Research, Guangdong Medical University, Zhanjiang 524023, China

**Keywords:** latent autoimmune diabetes in adults, human leukocyte antigen, DQB1, DRB1, polymorphisms, meta-analysis, relative predispositional effects

## Abstract

Latent autoimmune diabetes in adults (LADA) was recently demonstrated to be the most frequent form of adult-onset autoimmune diabetes mellitus. Case–control studies have investigated the relationship between human leukocyte antigen (HLA)-DQB1 and HLA-DRB1 polymorphisms and LADA risk, but their conclusions are inconsistent. This study aimed to more precisely explore the correlation between these HLA gene variants and LADA development. Eight databases, including PubMed, Embase, and Medline, were systematically searched for relevant studies up to September 15, 2018. We performed this retrospective study using meta-analysis and relative predispositional effect (RPE) methods. The meta-analysis results indicated that DQB1*02 (odds ratio (OR) = 1.685, *p_c_* < 0.005) and DQB1*06 (OR = 0.604, *p_c_* = 0.010) have opposite effects on susceptibility to LADA, while a significant decrease in LADA risk caused by DQB1*05 (OR = 0.764, *p_c_* = 0.100) disappeared upon Bonferroni correction. The RPE method confirmed the roles of DQB1*02 (χ² = 46.475, *p* < 0.001) and DQB1*06 (χ² = 17.883, *p* < 0.001) and further suggested protective effects of DQB1*05 (χ² = 16.496, *p* < 0.001). Additionally, the meta-analysis results showed that DRB1*03 (OR = 2.685, *p_c_* < 0.013), DRB1*04 (OR = 1.954, *p_c_* < 0.013), and DRB1*09 (OR = 1.346, *p_c_* < 0.013) are associated with increased LADA risk, while DRB1*12 (OR = 0.600, *p_c_* < 0.013) and DRB1*13 (OR = 0.583, *p_c_* < 0.013) carriers have a decreased risk of developing LADA. Furthermore, the RPE method revealed that DRB1*03 (χ² = 98.754, *p* < 0.001), DRB1*04 (χ² = 94.685, *p* < 0.001), DRB1*09 (χ² = 40.489, *p* < 0.001), DRB1*01 (χ² = 12.181, *p* < 0.001), DRB1*07 (χ² = 10.882, *p* = 0.001), and DRB1*08 (χ² = 5.000, *p* = 0.025) play protective roles against LADA. LADA showed a close relationship with genetic polymorphisms of HLA-DQB1 and WHLA-DRB1, which could contribute to a better understanding of disease pathogenesis and the identification of predisposing loci in the diagnosis and treatment of LADA.

## 1. Introduction

Latent autoimmune diabetes in adults (LADA) is an autoimmune disease characterized by onset in adults and the presence of diabetes-associated autoantibodies but no requirement for insulin treatment for a period (usually six months) after diagnosis [1]. In 1999, the World Health Organization (WHO) attributed LADA to type 1 diabetes mellitus (T1DM) [2] because both of them present impairment of pancreatic islet β-cells and autoantibodies (usually positive glutamic decarboxylase-65 antibody (GADA positive)) [3]. However, LADA shares similar clinical characteristics with type 2 diabetes mellitus (T2DM), such as onset in adulthood, increased likelihood in overweight patients, and association with rare ketosis as well as an increased risk of metabolic syndrome, such that it is often misdiagnosed as T2DM [4]. The morbidity of LADA among patients with newly diagnosed type 2 diabetes ranges from 5.9% to 9.2% in China [5] and 4 to 14% in Northern Europe [1], which might prevent the best opportunity for correct treatment and intervention. Improving knowledge of the mechanisms underlying LADA will have applications for diagnosis and therapy. Although the exact etiology of LADA is still not completely understood, there is growing evidence that the interaction between environmental factors and genetic factors contributes to the risk of LADA. For example, LADA susceptibility is associated with variants in genes including PTPN22 [6], CTLA4 [7], SH2B3 [8], and HLA [9].

The major histocompatibility complex (MHC) is a group of genes located on the sixth chromosome that encode the human leukocyte antigen (HLA) system (IMGT/HLA Database: http://www.ebi.ac.uk/imgt/hla). The *HLA* system is divided into the class I, II, and III regions. The *HLA* class II region consists of nearly 30 genes, including *HLA-DQB1*, *HLA-DQB2*, *HLA-DRA*, *HLA-DRB1*, *HLA-DRB2*, *HLA-DRB3*, *HLA-DRB4*, *HLA-DRB5*, *HLA-DQA1*, *HLA-DQA2*, *HLA-DPA1*, and *HLA-DPB1* as well as less variable genes involved in antigen processing and presentation [10]. The HLA class II region is critical in mediating the humoral immune response [11]. Among the genes mentioned above, *HLA-DQB1* and *HLA-DRB1* are associated with many immunological diseases, such as pemphigus vulgaris [12], narcolepsy [13], Alzheimer’s disease [14], and dermatomyositis [15].

Over the past few decades, many studies have reported that the *HLA-DQB1* and *HLA-DRB1* genes are related to LADA. Pan X. et al. first proposed that the nonaspartic acid homozygote gene at position 57 of *HLA-DQB1* is associated with LADA susceptibility in Chinese populations [16]. In previous studies, the allele groups of this gene, including HLA-DQB1*02 [17,18,19,20], HLA-DQB1*05 [19], HLA-DQB1*06 [19,20,21], HLA-DRB1*03 [19,20,21,22,23], HLA-DRB1*04 [19,20,21,22,24], HLA-DRB1*07 [21], HLA-DRB1*08 [20,21], HLA-DRB1*09 [20], HLA-DRB1*12 [19,20,21], and HLA-DRB1*13 [19], were reported to be associated with LADA. However, contradictory conclusions were drawn regarding these HLA allele groups. For example, DQB1*02 was not associated with LADA risk in four previous studies of European populations [21,22,23,24], but the data from four other studies, including one European study and three Asian studies, indicated that DQB1*02 increases susceptibility to LADA [17,18,19,20]. DQB1*06 was shown to have a protective effect against LADA in three studies, including one of Asians [20] and two of Europeans [19,21], yet another four studies, including two European studies [23,24] and two Asian studies [17,18], did not find any association. DRB1*03 was found to increase susceptibility to LADA in four European studies [19,21,22,23] and one Asian study [20], but its effect was not significant in the European population study by Vatay A. et al. DRB1*04 was reported in five studies, including four among Europeans and one among Asians, to play a role in the risk of developing LADA [19,20,21,22,24], but Cejkova P. et al. [23] reported no significant correlation between DRB1*04 and LADA among Europeans.

The previous conclusions drawn regarding the correlations between these genetic polymorphisms and LADA are inconsistent, possibly due to the small sample sizes used in the individual studies. In addition, when strong associations are found between certain alleles and LADA, it may be difficult to determine whether an allele is truly protective or if the observed effect is actually the result of another allele that has a stronger impact. Less effective alleles can also be masked by stronger alleles in a similar way. These effects have probably led to the contradictory conclusions in previous studies on *DQB1* and *DRB1*. The relative predispositional effect (RPE) method, which can identify the sequential association between alleles and LADA, may clarify the actual associations between alleles and LADA, regardless of the strength of the associations [25].

No meta-analysis or systematic review has provided a precise assessment of the relationships between *HLA-DQB1* and *HLA-DRB1* polymorphisms and LADA. We performed this study to determine these relationships by combining meta-analysis and RPE methods. Our aim was to provide a better understanding of the etiology and pathogenesis of LADA as well as the assessment and diagnosis of LADA in order to predict high-risk LADA patients with polymorphisms of these two *HLA* genes.

## 2. Materials and Methods

### 2.1. Search Strategy and Selection Criteria

We searched the PubMed, Embase, Medline, Web of Science, CNKI (China National Knowledge Infrastructure), Wanfang, VIP (China Science and Technology Journal Database), and SinoMed databases for relevant studies published on or before September 15, 2018. The following key words were used to search the above databases: (Latent Autoimmune Diabetes in Adults OR Diabetes Mellitus Type 1.5 OR Type 1.5 Diabetes Mellitus OR Type 1.5 Diabetes OR Diabetes, Type 1.5 OR LADA, Latent Autoimmune Diabetes in Adults OR Latent Autoimmune Diabetes of Adults) AND (*HLA-DQB1* OR *HLA-DRB1*). The studies included in this meta-analysis met the following criteria: I) they included a case–control design and human subjects; II) they examined the association between LADA risk and variants in the *DRB1* or *DQB1* locus; and III) they provided sufficient allele group data for *DRB1* or *DQB1* among both cases and controls to calculate odds ratios (ORs). Here, the patients with LADA were defined to comply with the following: I) adult onset (no younger than 18 years); II) no insulin treatment after diagnosis (at least three months); and III) positive for islet autoantibodies (antiglutamic acid decarboxylase antibody (GADA), islet antigen 2A (IA-2A) or antibodies against islet cells (ICAs)). Papers that did not satisfy the above inclusion criteria were excluded from our meta-analysis.

### 2.2. Data Extraction and Quality Assessment

The following information about the eligible studies was extracted: I) the name of the first author and the year of publication; II) the country or area in which the study was conducted and the numbers of cases and controls; and III) the age at onset, duration of diabetes, LADA diagnostic criteria (onset age, time without insulin intervention after diagnosis, and status of the islet autoantibodies), and HLA genotyping methods. We used the Newcastle–Ottawa Scale (NOS) to evaluate the methodological quality of the included studies. Studies scoring six points or more were considered high quality.

### 2.3. Statistical Analysis

Odds Ratios with 95% confidence intervals (CIs) were used to measure the effect strength of DQB1 or DRB1 on LADA susceptibility. For the studies [17,18,19,20,21,22,23,24,26] that reported ORs separately for the associations between LADA and the polymorphisms in the *HLA-DRB1* or *DQB1* allele groups (e.g., the HLA-DQB1*05 allele group includes the HLA-DQB1*05:01, HLA-DQB1*05:02, HLA-DQB1*05:03, and HLA-DQB1*05:04 alleles, among others, and in the study by Desai M. et al., the frequencies of these four alleles in the HLA-DQB1*05 allele group in patients with LADA and healthy controls were reported [19]), we combined the alleles into appropriate groups and calculated the combined ORs using a fixed-effects model (the Mantel–Haenszel method) for the main analysis [27]. The appropriate effect models were then selected to estimate the overall strength of the effects of the polymorphisms of each *HLA-DQB1* or *HLA-DRB1* allele group on LADA risk across studies based on a heterogeneity test. Heterogeneity was evaluated according to the *p_h_*-value of the χ^2^-based *Q* (Cochran’s *Q* test) and *I^2^* test statistics [28]. If *I^2^* < 50%, then the fixed-effect model (the Mantel–Haenszel method) [27] was applied. Otherwise, we selected the random-effects model (the DerSimonian–Laird method) [29]. *I^2^*-values < 25%, 25% ~ 50%, 50% ~ 75%, and > 75% represent no, low, moderate, and high heterogeneity, respectively [28]. The Bonferroni method was used to correct the *p*-values of the *Z*-test (*p_c_* = *p* * *n*, where *n* represents the number of alleles in *HLA-DQB1* or *HLA-DRB1* chains involved in our study) [30].

Power and Sample Size Calculation (PS) version 3.1.2 (http://biostat.mc.vanderbilt.edu/wiki/Main/PowerSampleSize) [31] was used to perform power calculations for the association between the polymorphisms of each allele group of the *HLA-DQB1* or *HLA-DRB1* gene and LADA, and a value greater than 0.8 indicated a high degree of statistical power in this meta-analysis. We also performed a sensitivity analysis by removing one study at a time to assess whether the final result was strongly affected by one study. Funnel plot analysis and Begg’s test or Egger’s test were conducted to assess publication bias. Subgroups stratified by ethnicity were used to study the effects of the *HLA-DQB1* and *HLA-DRB1* allele groups on LADA risk across different populations. In the meta-regression analysis, we explored the sources of heterogeneity, including the year of publication, population, NOS score, and number of participants in the included studies. Here, the RPE method was used to analyze the allelic data for other allele groups to avoid skewed results due to a strongly associated allele group. In the RPE method, an χ² test was used to detect the overall frequency contribution of each allele or each allele group in order to find the allele or allele group with the largest deviation between the observed and expected frequencies [25]. The strongest associated allele or allele group identified in each round was removed in the next round of comparison. The procedure was sequentially repeated until no significant deviation of the overall contribution was observed. Standardization was used to correct the observed value obtained by the RPE method, which may prevent erroneous results due to different sample sizes of various alleles [25]. All analyses were performed using STATA version 14.0 software (STATA Corporation, College Station, TX, USA) with the “metan” command [32].

## 3. Results

### 3.1. Literature Search Results and Characteristics of the Included Studies

**Figure 1:**
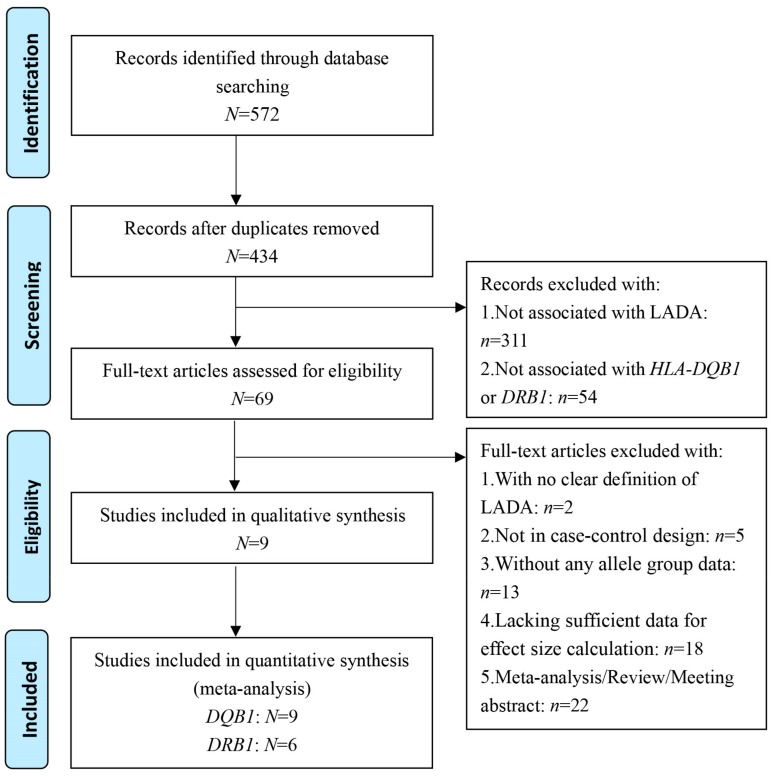
Flow diagram of the process used to select eligible studies.

Figure 1 shows the literature search and study selection procedure used in this work. The initial search strategy yielded 572 potential records on or before September 15, 2018. A total of 138 duplicates were removed, leaving 434 articles. After careful examination of the titles and abstracts of these remaining articles, 365 were excluded: 311 articles not associated with LADA disease and 54 articles not related to the *HLA-DQB1* and *HLA-DRB1* genes. Subsequently, we further screened the remaining records (*N* = 69) and removed two articles with no clear definition of LADA, five articles without a case–control design, 13 articles without any allele group data, 18 articles lacking sufficient data necessary for effect size calculation, and 22 articles classified as a meta-analysis, review, or meeting abstract. Finally, nine articles with a total of 1324 patients with LADA and 2657 healthy controls were included in this meta-analysis. Among these studies, nine were on *DQB1*, with 1278 cases and 2581 controls, and six were on *DRB1*, with 1197 cases and 2139 controls.

Table 1 presents the main characteristics of the nine studies included in this meta-analysis. These studies were published from 1997 to 2017. Among the studies, six were conducted on individuals of European origin [19,21,22,23,24,26], and the other three were conducted on Asian populations [17,18,20]. Additionally, six articles provided case–control data regarding both *DQB1* and *DRB1* polymorphisms and LADA, while three articles reported only the correlations between *DQB1* variants and LADA. The age at onset of patients with LADA ranged from 20 to 82 years old; the duration of the disease reported in six articles ranged from newly diagnosed to 32 years [18,19,21,22,23,26], while three studies [17,20,24] did not report this variable. Moreover, the following three *HLA* genotyping methods were used in the original studies: single specific primer-polymerase chain reaction (SSP-PCR) in seven studies [18,19,21,22,23,24,26], PCR-sequence-based typing (SBT) in one study [20], and PCR/sequence-specific oligonucleotide (SSO) typing in one study [17]. The NOS results indicated that all eligible studies were of high quality since their scores were greater than or equal to six points (Table 1 and Table S1).

We also compared the characteristics of LADA patients with those of controls, including the ratio of males to females, mean age and body mass index (BMI), and islet autoantibody detection. Four articles reported the BMI of LADA patients. The mean BMI ranged from 23.5 to 31.6 kg/m², in which the minimum was close to the upper limit of a normal BMI (23.9 kg/m²) and the maximum was slightly higher than the threshold for obesity (30.0 kg/m²), suggesting a BMI scope of LADA between that of T1DM (lower) and T2DM (higher). Additionally, eight articles [17,18,20,21,22,23,24,26] stated that the LADA patients did not receive insulin treatment for six months after diagnosis, while in the research of Desai M. et al. [19], the periods without insulin treatment after diagnosis for the LADA patients from the UK Prospective Diabetes Study (UKPDS), the Warren 2 Repository (W2), and the Exeter Young-Onset Type 2 Diabetes Study (YT2D) were three, twelve, and three months, respectively. Regarding disease detection, LADA patients in all included studies were at least GADA positive and were shown to have autoantibody positivity for IA-2A and ICAs in the study by Hosszufalusi N. et al. [26], for IA-2A in the YT2D study by Desai M. et al. [19] and the studies by Xiao J.Z. et al. [17], Yin N.N. et al. [20], and Cerna M. et al. [21], and for ICAs in the study by Vatay A. et al. [24] (Table S2).

### 3.2. Association between HLA-DQB1 Polymorphisms and Susceptibility to Latent Autoimmune Diabetes in Adults

#### 3.2.1. A Meta-Analysis of the Association between *HLA-DQB1* Polymorphisms and Susceptibility to Latent Autoimmune Diabetes in Adults 

The associations of *HLA-DQB1* polymorphisms with the risk of LADA found in this meta-analysis are detailed in Table 2. The frequency of DQB1*02 was clearly higher in patients with LADA than in the healthy controls (OR = 1.685, 95% CI: 1.298–2.187, *p_c_* < 0.005), indicating that the DQB1*02 allele group is a strong risk factor for LADA. DQB1*06 showed a significant association with a decreased risk of LADA (OR = 0.604, 95% CI: 0.438–0.833, *p_c_* = 0.010), revealing a protective role of this allele group against the development of LADA. Additionally, the high statistical power of these associations reveals the robustness of this result (DQB1*02: *p_power_* = 1.000; DQB1*06: *p_power_* = 0.928). In contrast, DQB1*05 had higher frequencies in controls than in patients with LADA, suggesting that DQB1*05 has a protective effect against LADA (DQB1*05: OR = 0.764, 95% CI: 0.609–0.958, *p* = 0.020). However, the difference was not significant after the *P*-values were corrected by the Bonferroni method (DQB1*05: *p_c_* = 0.100). Furthermore, no association was found between the frequency of DQB1*03 or DQB1*04 and the risk of LADA (DQB1*03: *p* = 0.296 and DQB1*04: *p* = 0.616). In the analysis of population-origin subgroups, DQB1*02 showed a significant correlation with LADA development among Asian populations (OR = 2.312, 95% CI: 1.873–2.854, *p_c_* < 0.005), but the CIs slightly overlapped in the European populations (OR = 1.387, 95% CI: 0.987–1.950, *p* = 0.059). In contrast, DQB1*06 was significantly associated with a lower risk of LADA in European populations (OR = 0.430, 95% CI: 0.346–0.535, *p_c_* < 0.005) but not in Asian populations (*p* = 0.990). Again, DQB1*05 showed a protective effect against LADA (OR = 0.769, 95% CI: 0.612–0.964, *p* = 0.023) but not after Bonferroni correction (*p_c_* = 0.115) in the European populations, while it was not associated with this disease in the Asian populations (OR = 0.286, 95% CI: 0.015–5.463, *p* = 0.406). Additionally, DQB1*03 and DQB1*04 were not associated with the risk of LADA in either ethnicity group (Table 3).

According to the results of the homogeneity test (Table 2), low heterogeneity among studies was observed for DQB1*05 (*p_h_* = 0.182, *I^2^* =35.9%), while a moderate variation across studies was found for DQB1*02 (*p_h_* = 0.006, *I^2^* = 64.3%), DQB1*03 (*p_h_* = 0.013, *I^2^* = 58.8%), DQB1*04 (*p_h_* = 0.025, *I^2^* = 61.0%), and DQB1*06 (*p_h_* = 0.006, *I^2^* = 66.7%). Meta-regression analysis revealed that the heterogeneity among studies on DQB1*04 resulted from differences in research quality (*p-regression* = 0.028). However, the sources of heterogeneity across the studies investigating other allele groups were not detected in the meta-regression (Table S3).

**Figure 2:**
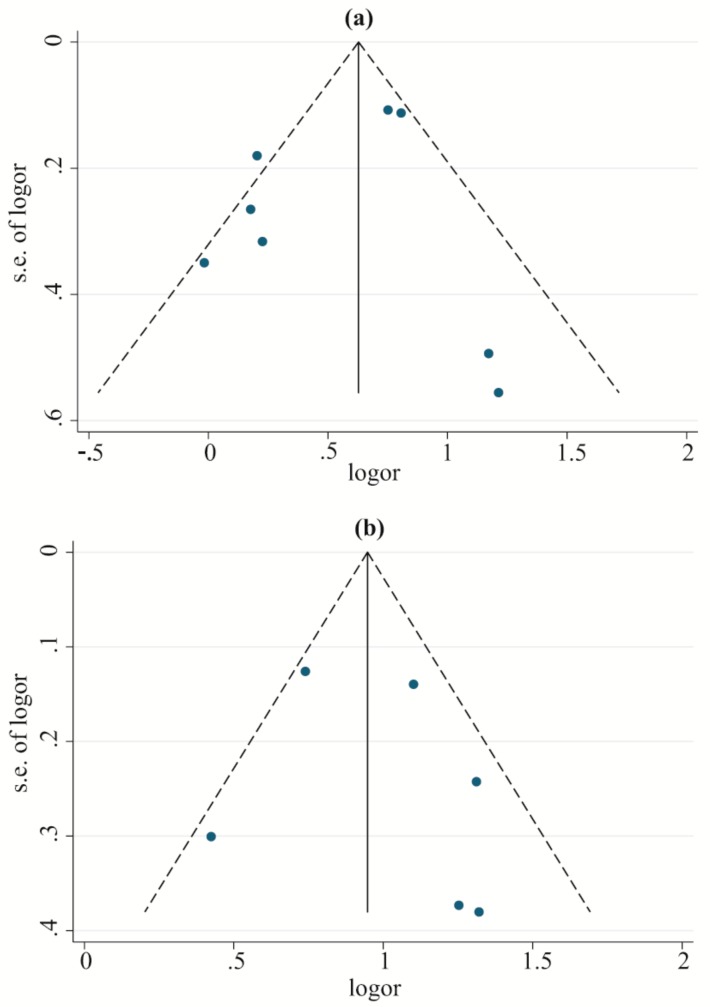
Funnel plots to analyze publication bias for the associations between the HLA-DQB1*02 and HLA-DRB1*03 polymorphisms and the risk of LADA: (**a**) DQB1*02; (**b**) DRB1*03. Note: s.e.: standard error.

In a leave-one-out sensitivity analysis, the results showed that the association between DQB1*05 and the risk of LADA became statistically nonsignificant after removing the study by Desai M. et al. (2007) or that by Cerna M. et al. (2003) (Table S4). The pooled ORs were not significantly different for other variants in *DQB1*. The funnel plot, Begg’s test, and Egger’s test indicated no obvious publication bias in the included studies (Table S5). Figure 2a illustrates no publication bias for the association of the HLA-DQB1*02 polymorphism with LADA risk.

#### 3.2.2. Relative Predispositional Effect of *HLA-DQB1* Polymorphisms on the Risk of Latent Autoimmune Diabetes in Adults

Table 4 shows the results of the RPE method for the association between *DQB1* and LADA. The observed frequencies of DQB1*02 were significantly higher than the expected values in patients with LADA (observed vs. expected = 583 vs. 440), suggesting that DQB1*02 is a risk factor for LADA, and this allele group was the strongest predisposing allele group that increased the risk of LADA in the first round (χ² = 46.475, *p* < 0.001). With DQB1*02 removed, we found that DQB1*06 was protective against the development of LADA (χ² = 17.883, *p* < 0.001). Removing DQB1*06 further suggested the protective effect of DQB1*05 (χ² = 16.496, *p* < 0.001). After removing DQB1*02, DQB1*06, and DQB1*05 sequentially, no remaining allele groups had a significant effect.

### 3.3. Association between HLA-DRB1 Polymorphisms and Susceptibility to LADA

#### 3.3.1. A Meta-Analysis of the Association between *HLA-DRB1* and Susceptibility to LADA

The results of the meta-analysis of the association between *HLA-DRB1* variants and susceptibility to LADA are shown in Table 5. The frequencies of DRB1*03 (OR = 2.685, 95% CI: 2.060–3.501, *p_c_*< 0.013), DRB1*04 (OR = 1.954, 95% CI 1.507–2.535, *p_c_* < 0.013), and DRB1*09 (OR = 1.334, 95% CI: 1.141–1.559, *p_c_* < 0.013) were significantly higher in patients with LADA than in the controls, which suggests that all three are risk factors for LADA. In contrast, DRB1*12 (OR = 0.600, 95% CI: 0.479–0.751, *p_c_* < 0.013) and DRB1*13 (OR = 0.583, 95% CI: 0.432–0.787, *p_c_* < 0.013) showed protective effects against LADA. In addition, DRB1*01, DRB1*07, DRB1*08, DRB1*10, DRB1*11, DRB1*14, DRB1*15, and DRB1*16 had no association with LADA. In the subgroup analysis by population origin, both DRB1*03 (Asian: OR = 2.095, 95% CI: 1.636–2.682, *p_c_* < 0.013 and European: OR = 2.942, 95% CI: 2.205–3.924, *p_c_* < 0.013) and DRB1*04 (Asian: OR = 1.886, 95% CI: 1.468–2.424, *p_c_* < 0.013 and European: OR = 2.049, 95% CI: 1.383–3.035, *p_c_* < 0.013) showed significant correlations with LADA development among Asian and European populations. In contrast, DRB1*12 had significant associations with a lower risk of LADA in both the Asian (OR = 0.632, 95% CI: 0.502–0.795, *p_c_*< 0.013) and European populations (OR = 0.215, 95% CI: 0.077–0.598, *p_c_* < 0.013). Additionally, DRB1*08 was significantly associated with a decreased risk of LADA in the Asian populations (OR = 0.574, 95% CI: 0.439–0.751, *p_c_* < 0.013) but not in the European populations (*p* = 0.390), while DRB1*09 was detected as a susceptibility factor for LADA in the Asian populations (OR = 1.359, 95% CI: 1.160–1.593, *p_c_* < 0.013) but not in the European populations (*p* = 0.904) (Table 6). A subgroup analysis was not conducted for the remaining allele groups because the studies of these groups involved only Europeans.

The heterogeneity analysis revealed no obvious heterogeneity in the studies on DRB1*01 (*p_h_* = 0.284, *I^2^* = 21.0%), DRB1*09 (*p_h_* = 0.369, *I^2^* = 1.8%), DRB1*10 (*p_h_* = 0.539, *I^2^* = 0.0%), DRB1*13 (*p_h_* = 0.938, *I^2^* = 0.0%), DRB1*14 (*p_h_* = 0.565, *I^2^* = 0.0%), and DRB1*16 (*p_h_* = 0.627, *I^2^* = 0.0%) and low heterogeneity in studies on DRB1*12 (*p_h_* = 0.157, *I^2^* = 39.6%); moderate heterogeneity was observed in studies on DRB1*03 (*p_h_* = 0.054, *I^2^* = 54.0%) and DRB1*04 (*p_h_* = 0.070, *I^2^* = 50.9%). In contrast, studies on DRB1*07 (*p_h_* = 0.003, *I^2^* = 78.9%), DRB1*08 (*p_h_* < 0.001, *I^2^* = 77.9%), DRB1*11 (*p_h_* = 0.001, *I^2^* = 82.4%), and DRB1*15 (*p_h_* = 0.001, *I^2^* = 82.6%) had high heterogeneity. In the meta-regression analysis, no factors were able to eliminate or decrease the heterogeneity to an acceptable level (all *p-regression* > 0.05, as shown in Table S3).

In the sensitivity analysis, the results showed a statistically significant protective role of DRB1*08 against LADA risk after removing the study by Cerna M. et al. (2003) (Table S4); for DRB1*09, the correlation with LADA development became statistically nonsignificant after removing the study by Yin N.N. et al. (2017) (Table S4). No significant changes in the ORs of other allele groups were found. In addition, no obvious publication bias of the included studies was found in the funnel plot, Begg’s test, or Egger’s test (Table S5). Figure 2b illustrates no publication bias for the association of the HLA-DRB1*03 polymorphism with LADA risk.

#### 3.3.2. Relative Predispositional Effect of *HLA-DRB1* Polymorphisms on the Risk of LADA

Table 7 displays the data from the RPE analysis of *DQR1* and LADA in detail. In the first round of comparison, the observed frequencies of DQR1*03 were significantly higher than the expected values in patients with LADA (observed vs. expected = 415 vs. 256), revealing that DRB1*03 results in the strongest susceptibility to LADA (χ² = 98.754, *p* < 0.001). After the removal of DRB1*03, DRB1*04 was also found to predispose patients to the disease (χ² = 94.685, *p* < 0.001). With DRB1*04 removed, the expected DRB1*09 frequencies were significantly lower than the observed frequencies, suggesting an effect of this allele group on the susceptibility to LADA (χ² = 40.489, *p* < 0.001). The frequencies of DRB1*01 were lower in the controls than in LADA patients after the third round, indicating an effect on the risk of developing LADA (χ² = 12.181, *p* < 0.001). After removing DRB1*01, DRB1*07 was found to be associated with susceptibility to LADA (χ² = 10.882, *p* = 0.001). DRB1*08 was also shown to be associated with susceptibility to LADA after the removal of DRB1*07 (χ² = 5.000, *p* = 0.025). No allele groups were found to have a significant effect after removing DRB1*03, DRB1*04, DRB1*09, DRB1*01, DRB1*07, and DRB1*08 sequentially (Table 7).

### 3.4. Association of DQB1 or DRB1 Polymorphisms with LADA Risk Based on Two Methods

In summary, the results from both the meta-analysis and the RPE method indicated that the allele groups HLA-DQB1*02, HLA-DQB1*06, HLA-DRB1*03, HLA-DRB1*04, and HLA-DRB1*09 increased susceptibility to LADA, while HLA-DQB1*03, HLA-DQB1*04, HLA-DRB1*10, HLA-DRB1*11, HLA-DRB1*14, HLA-DRB1*15, and HLA-DRB1*16 did not have an effect on the development of the disease. In addition, comparing the results of these two methods revealed that HLA-DQB1*05 had a protective effect against LADA, while HLA-DRB1*01, HLA-DRB1*07, and HLA-DRB1*08 were associated with susceptibility to the disease according to the RPE method but not the meta-analysis. However, it is worth noting that, without Bonferroni *P*-value correction, HLA-DQB1*05 was also a protective factor against LADA in the meta-analysis. In contrast, DRB1*12 and DRB1*13 were protective allele groups according to the meta-analysis but not the RPE method. As a result, HLA-DRB1*01, HLA-DRB1*07, HLA-DRB1*08, HLA-DRB1*12, and HLA-DRB1*13 may have an association with the risk of developing LADA, but they need to be further examined.

## 4. Discussion

LADA is an autoimmune disease, the pathogenesis of which is currently unclear but is likely the result of environmental factors interacting with genetic susceptibility. Studies have been conducted on the association between *HLA-DQB1* or *HLA-DRB1* polymorphisms and the risk of LADA, but their outcomes were conflicting. On the one hand, although LADA is the most frequent form of adult-onset autoimmune diabetes [1], the diagnosis of LADA is still a challenge in clinics [4], leading to difficulty in the collection of data regarding related genes. The inconsistent results from previous studies seem to be mainly due to the small sample sizes used in the studies leading to low statistical power. On the other hand, *HLA* is a highly heterogeneous gene, with 1142 and 2103 alleles in the *DQB1* and *DRB1* regions, respectively [33], and easily misinterpreted variation in the frequency of some alleles because of the presence of a strong disease-associated allele or allele frequencies that are too low to calculate [34]. Thus, we used meta-analysis to integrate the results of independent studies and employed the RPE method to sequentially detect associated allele groups in order to minimize deviation. Briefly, the combination of meta-analysis and RPE methods can determine the relations between LADA and *HLA-DQB1* or *HLA-DRB1* polymorphisms more accurately and more consistently.

**Figure 3:**
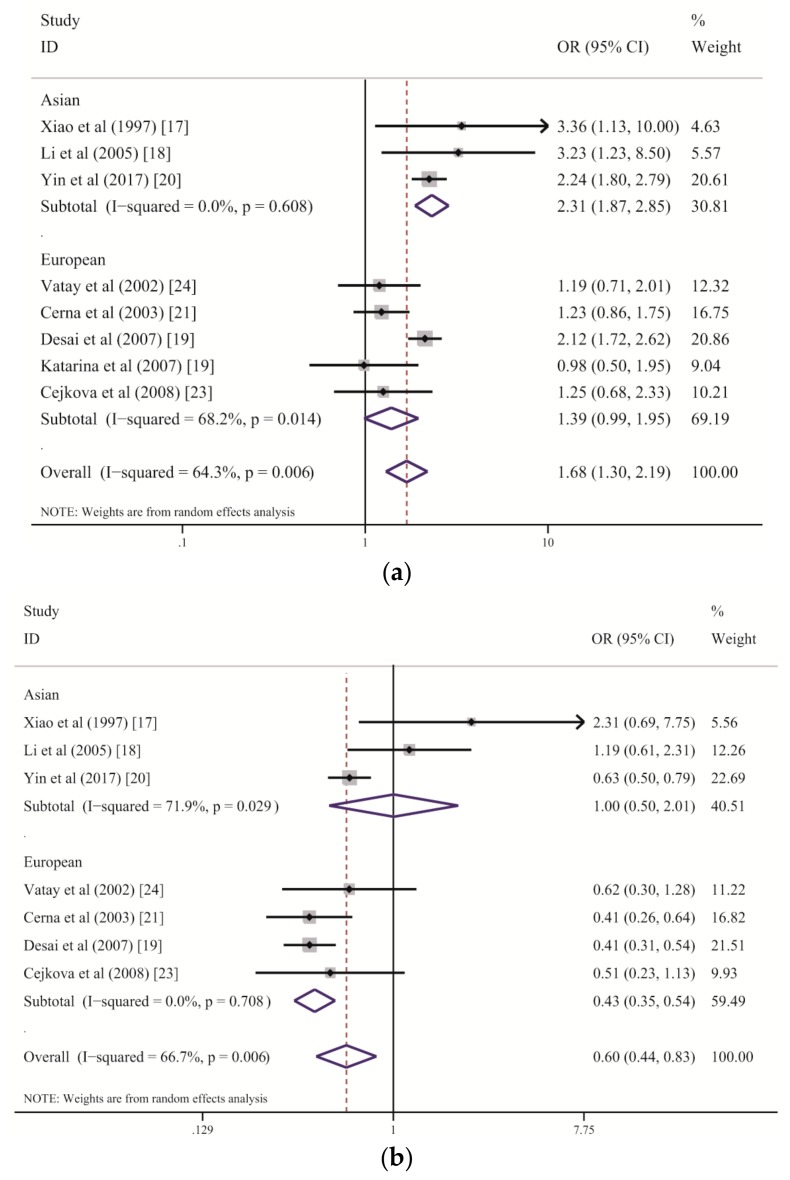
Forest plots of the subgroup analysis by ethnicity for correlations between HLA-DQB1*02 and HLA-DQB1*06 variants and the risk of LADA: (**a**) DQB1*02; (**b**) DQB1*06.

Consistent results from both the meta-analysis and RPE methods indicate that some allele groups of the *HLA-DQB1* gene confer strong susceptibility to LADA. In our study, HLA-DQB1*02 was demonstrated to be the main allele group in the *DQB1* region predisposing patients to LADA, although moderate heterogeneity was found among the included studies (Table 2). The subgroup analysis stratified by ethnicity suggested that DQB1*02 increases the risk of LADA among the Chinese Han population but has only a marginal association with LADA among people of European descent (Figure 3a and Table 3). Genetic and humoral heterogeneity between LADA and HLA genes were also found in previous studies. In Chinese studies [17,18,20], all correlations between DQB1*02 and the risk of LADA were consistent (Figure 3a). In contrast, three of five eligible studies of European populations demonstrated slightly higher frequencies of DQB1*02 among patients with LADA than among the controls but with wide CIs that marginally crossed one [21,23,24]; the study by Desai M. et al. was weighted the heaviest and displayed a significant association [19]. These results suggest that larger sample sizes are required to detect the role of DQB1*02 among Europeans because its effect strength in European populations is weaker than that in Chinese populations. In fact, the combined ORs suggest that the DQB1*02 allele group confers a two-fold higher risk of LADA in the Chinese populations than in the European populations (Chinese: OR = 2.31; European: OR = 1.39). We further analyzed DQB1*02:01 separately (7 of 8 studies) and found a significant association of this allele with the risk of LADA (OR = 2.015, 95% CI: 1.459–2.784, *p* < 0.001, not shown in the tables). The above results suggest that HLA-DQB1*02 is a stable allele group predisposing patients to LADA, although its effect strength varies across ethnicities.

In contrast to the predisposing effect of DQB1*02 with regard to LADA, DQB1*06 within the *HLA-DQB1* gene plays a significantly protective role, as revealed by both the meta-analysis and RPE methods, although a high level of heterogeneity was found among studies (Table 2 and Figure 3b). The subgroup analysis stratified by ethnicity demonstrated an association between decreased susceptibility to LADA and DQB1*06 in the European populations, with no heterogeneity, but there was no effect of this allele group on the disease in the Chinese populations, with a high degree of between-study heterogeneity (Table 3 and Figure 3b). We suspect that the variance among populations is primarily due to environmental differences and the effect strength of DQB1*02. DQB1*06 showed a strong protective role against LADA among the European populations because of the relatively weak role played by DQB1*02; however, its protective effect was substantially reduced by the stronger effect of DQB1*02 among the Chinese populations. This finding was also confirmed by the RPE analysis. Consequently, DQB1*06 is an allele group that confers resistance to LADA but that might be influenced by DQB1*02.

**Figure 4:**
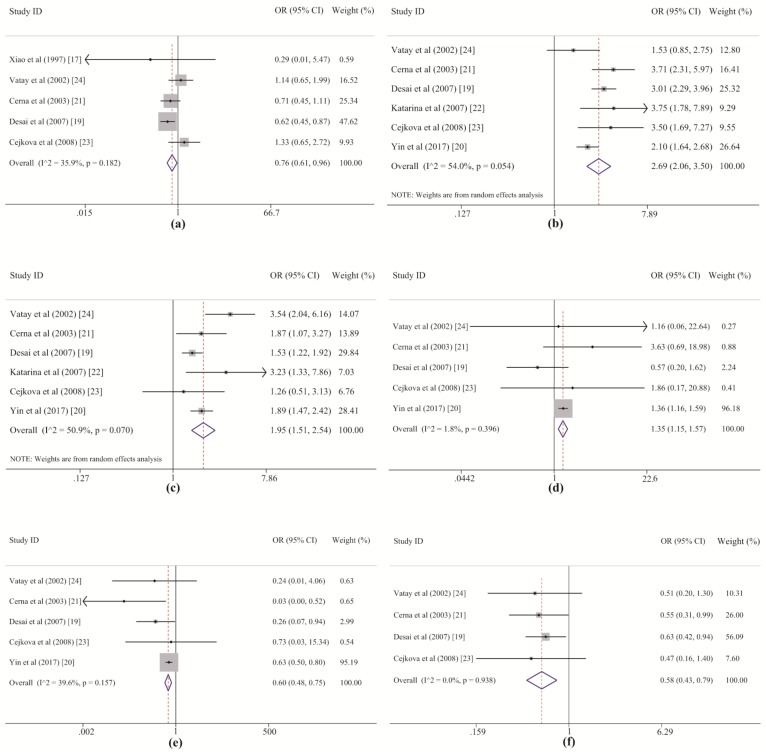
Forest plots of the associations between the HLA-DQB1*05, HLA-DRB1*03, HLA-DRB1*04, HLA-DRB1*09, HLA-DRB1*12, and HLA-DRB1*13 polymorphisms and the risk of LADA: (**a**) DQB1*05; (**b**) DRB1*03; (**c**) DRB1*04; (**d**) DRB1*09; (**e**) DRB1*12; (**f**) DRB1*13.

Our meta-analysis initially suggested a protective effect of DQB1*05 against LADA, but this effect disappeared after Bonferroni correction. RPE analysis further showed the protective effect of this allele group after removing DQB1*02 and DQB1*06. Thus, DQB1*05 might play a protective role through an alternate pathway that is independent of DQB1*02 and DQB1*06. With regard to the DQB1*05 allele group, the heterogeneity between studies was at an acceptable level (Table 2 and Figure 4a). The dataset from the study by Desai M. et al. (2007) [19] revealed a correlation between LADA and DQB1*05 among Europeans, but no statistically significant associations were found in the other studies, including three in Europeans [21,23,24] and one in Asians [17]. Additionally, no effects of DQB1*05 alleles on the risk of LADA were detected in previous studies [17,19,21]. These prior studies may have failed to demonstrate a correlation between LADA and DQB1*05 because the weak effect of the allele group led to low statistical power for the test and was easily masked by the strong effect of DQB1*02. This point is also supported by the results of the sensitivity analysis, which suggested that this association was dependent on the two most heavily weighted studies (Table S4 and Figure 4a). Therefore, we conclude that DQB1*05 likely plays a protective role against the development of LADA, but its effect is weak and easily masked by the stronger effects of DQB1*02 and DQB1*06.

In this systematic study, DRB1*03, DRB1*04, and DRB1*09 within the *HLA-DRB1* gene were also demonstrated to strongly confer susceptibility to LADA in both the meta-analysis and the RPE analysis, and DRB1*03 was revealed as the allele group in the *DRB1* region with the strongest effect on LADA. For DRB1*03 and DRB1*04, the heterogeneity among the included studies was acceptable. Five of six of the original datasets showed good agreement with our results, and only the studies of European populations by Vatay A. et al. (2002) [24] and Cejkova P. et al. [23] respectively displayed marginal associations since their individual ORs were larger than one but their individual CIs crossed one slightly (Table 5 and Figure 4b,c). In the subgroup analysis by ethnicity, both DRB1*03 and DRB1*04 increased susceptibility to LADA in Asian and European populations, but high heterogeneity was observed for DRB1*04 among five studies in Europeans (Table 6). The highest statistical power (value of one) was detected in the associations between the risk of LADA and the DRB1*03 and DRB1*04 frequencies, indicating that these results are robust. For DRB1*09, only minor heterogeneity was found among the included studies (Table 5). DRB1*09 showed a significant association with an increased risk of LADA in the study by Yin N.N. et al. (2017) [20] among Asian populations (*p* < 0.001, *p_c_* < 0.013) but not among individuals of European origin (*p* < 0.904), for which none of four studies revealed a statistically significant association with the disease [19,21,23,24] (Table 6 and Figure 4d). The sensitivity analysis also revealed that the significance of the association changed after removing Yin et al.’s study. In addition, it is worth noting that the frequency of DRB1*09 was much lower in the European populations than in the Asian populations (European: 0.010; Asian: 0.226, shown in Table S6) and that the total numbers of cases and controls from the Asian study were even larger than those from four European studies (LADA: Asian: 652; European: 518; controls: Asian: 1171; European: 869, which can be derived from Table 1) in this meta-analysis. Therefore, based on the above results, three conclusions can be drawn. First, DRB1*09 is an allele group that predisposes Asians, especially Chinese populations, to LADA, even though only one study was performed to investigate their association. Second, DRB1*09 may not play a role in the disease for Europeans. Third, we prefer to consider the positive association rather than the negative association between this allele group and the risk of LADA in the overall population because the former association had a statistical power of 0.723 that was further confirmed by RPE analysis. However, these conclusions should be treated with caution, and further studies with larger sample sizes should be conducted to verify them in the future.

The meta-analysis and RPE analysis also led to contrasting conclusions. In the meta-analysis, both DRB1*12 and DRB1*13 conferred protection against LADA. For DRB1*12, three studies (one by Yin N.N. et al. of an Asian population and one each by Cerna M. et al. and Desai M. et al. of European populations) reported a protective role for DRB1*12 [19,21], but the other two European datasets from Cejkova P et al. and Vatay A et al. showed negative results (Figure 4e). However, although the associations could not be demonstrated in these two studies, because the frequencies were <5% in both the LADA and control groups [23,24], the OR for DRB1*12 was still lower than one, indicating a possible protective role (Figure 4e). For DRB1*13, allelic data from the studies of European populations by Cerna M. et al. and Desai M. et al. also showed significantly lower susceptibility to LADA among DRB1*13 carriers than among noncarriers; in contrast, although the other two European studies by Cejkova P et al. and Vatay A et al. also showed a strong protective role of DRB1*13, with very small ORs (0.47 and 0.51), the effects were not statistically significant (Figure 4f). The results of the meta-analysis for these two allele groups reinforced their protective role in LADA, indicating the advantage of the method, by which it is possible to properly evaluate real genetic effects on disease development with greater statistical power than that afforded by individual analyses pooling all samples or synthesizing the overall data available in previous studies. However, the results of the RPE analysis suggested that the protective effects of DRB1*12 and DRB1*13 were somewhat dependent on the susceptibility conferred by DRB1*03 and DRB1*04. Regardless of whether this dependence exists, the understanding of the associations between DRB1*12 and DRB1*13 and the risk of LADA in addition to the interactions between the different alleles will benefit from further studies with larger sample sizes and more haplotype data.

There were also contradictory results from the meta-analysis and the RPE analysis with regard to the associations of LADA with DRB1*01, DRB1*07, and DRB1*08. The meta-analysis suggested DRB1*01 and DRB1*07 have protective effects against LADA, with marginal or no statistical significance (Table 5); however, the RPE analysis indicated roles played by DRB1*01 and DRB1*07 in susceptibility to LADA in the fourth and sixth rounds, respectively. These relationships may be explained by the relatively weak effect of DRB1*01 and DRB1*07 on the risk of LADA compared with that of DRB1*03, DRB1*04, and DRB1*09. Based on the above results, DRB1*01 and DRB1*07 might promote the development of LADA. Thus, these substantially different results from the RPE method compared to those from meta-analysis or the individual studies might reflect the advantages of RPE analysis, which allows us to sequentially detect the associated alleles or allele group and minimize the variation caused by some specific loci or locus groups. Although the results from RPE analysis suggested that DRB1*08 increased disease risk, the pooled results of the meta-analysis showed no association with LADA, with a high degree of heterogeneity between studies. In contrast, in the sensitivity analysis, the results showed a protective role of DRB1*08 against the development of LADA after the European study by Cerna M. et al. [21] was removed, which also significantly reduced the heterogeneity (*I^2^* = 37.0%, shown in Table S4). In addition, the distinctive characteristics of cases and controls in individual studies, such as differences in the age of onset, varying degrees of disease development among patients, and dissimilarities in allele group frequency distributions among regions, might explain this high heterogeneity. However, the currently available evidence is insufficient to indicate whether LADA is related to the frequency of DRB1*08. Consequently, the roles played by DRB1*01, DRB1*07, and DRB1*08 in the development of LADA still require more data for verification.

The above allele groups, including DQB1*02, DQB1*05, DQB1*06, DRB1*01, DRB1*03, DRB1*04, DRB1*07, DRB1*08, DRB1*09, DRB1*12, and DRB1*13, were found to be associated with LADA risk to some extent by the meta-analysis and/or RPE method in our study. The groups were also previously correlated with other types of autoimmune diseases. For example, DQB1*02 and DQB1*05 are significantly associated with a decreased risk and an increased risk of pemphigus vulgaris (PV), respectively [35]. DQB1*06 and DRB1*13 have a protective effect against rheumatoid arthritis (RA), while DRB1*01 and DRB1*04 have a detrimental effect [36,37,38]. DRB1*03 and DRB1*12 are respectively protective against or predisposing to autoimmune thyroid diseases (AITDs) [39]. DRB1*07 and DRB1*08 are significantly correlated with the development of type 2 and type 1 autoimmune hepatitis (AIH), respectively [40], and DRB1*08 also increases susceptibility to juvenile idiopathic arthritis (JIA) [41]. In addition, DRB1*09 was detected to be a risk factor for systemic lupus erythematosus (SLE) [42]. Notably, the effects of these allele groups on susceptibility to other types of autoimmune diseases were similar to those on susceptibility to LADA.

Finally, both the meta-analysis and the RPE method demonstrated a lack of correlations between the other HLA genetic allele groups and LADA. We did not find any associations of HLA-DQB1*03, HLA-DQB1*04, HLA-DRB1*10, HLA-DRB1*11, HLA-DRB1*14, HLA-DRB1*15, and HLA-DRB1*16 with the risk of LADA when using either method, although some studies reported that these polymorphisms may be related to other autoimmune diseases, such as multiple sclerosis (MS) [43], Henoch–Schönlein purpura (HSP) [44], and T1DM [45].

In the past, LADA was considered to be a form of adult-onset T1DM, with many clinical features similar to those of T2DM. However, some evidence indicates that there is likely a different pathological mechanism driving LADA than either T1DM or T2DM [4,46,47]. Furthermore, there is genetic, phenotypic, and humoral heterogeneity in the degrees of insulin resistance and autoimmunity. Comparing our conclusion with that of a previously published meta-analysis on the association between HLA class II alleles and T1DM in Latin American patients [45], these two diseases display some heterogeneity in their correlations with *HLA-DQB1* and *HLA-DRB1*. First, although DQB1*02, DRB1*03, and DRB1*04 increase an individual’s susceptibility to both LADA and T1DM, while DQB1*05, DQB1*06, and DRB1*13 protect an individual from developing LADA and T1DM, these loci clearly have a stronger impact on T1DM than on LADA (e.g., DRB1*13: LADA: OR = 0.583; T1DM: OR = 0.300 [45]). In addition, the allele groups conferring susceptibility to or protection against LADA and T1DM differ. For example, DRB1*09 increases susceptibility to LADA, but this variant is not significantly associated with T1DM. In contrast, DQB1*03 increases susceptibility to T1DM, while DRB1*14 and DRB1*15 have protective effects; there are no obvious correlations between these variants and LADA. Moreover, some particular allele groups have opposite effects on the risk of LADA and T1DM. Based on our conclusions, DRB1*12 decreases the risk of developing LADA; however, it was shown to increase the risk of T1DM [45]. The abovementioned results partly support the view that LADA might have a different etiology than T1DM. However, the variance observed in the associations of T1DM and LADA with *HLA-DQB1* and *HLA-DRB1* could also be due to differences in the inclusion criteria and study design (such as diagnostic criteria, gender, and genotyping method) as well as statistical power and ethnicity. Thus, this conclusion needs to be treated with caution, and more evidence is needed to verify our hypothesis in the future.

The limitations of this retrospective study warrant further discussion. First, in the meta-analysis, high between-study heterogeneity was detected in the association between some allele groups, such as DRB1*07 and DRB1*11, and the risk for LADA; however, we failed to find the source of this heterogeneity through meta-regression, subgroup analysis, and sensitivity analysis. Second, although we performed a thorough and meticulous search to identify all available literature, we obtained only nine studies that fit the eligibility criteria for this meta-analysis. We even contacted the authors of original studies lacking sufficient allele data, but we did not receive a reply. Third, the small sample size of several case–control studies included here is an unavoidable limitation of the meta-analysis, and more studies with larger sample sizes are needed to support future retrospective analysis. Fourth, data on the *HLA-DQB1* and *HLA-DRB1* alleles (with four digits and high resolution) have been provided in increasing the numbers of original studies, but the data are still too limited for further exploration of the relationships of these alleles with the risk of developing LADA. Fifth, previous studies also reported relationships between the *HLA-DQB1* and *HLA-DRB1* genotypes, haplotypes, and LADA, but it is difficult to systematically evaluate these relationships due to sparse data. Therefore, future research should focus on the interplay between the *HLA-DQB1* and *HLA-DRB1* genes as well as alleles (with four digits) and investigate the stability of their associations with LADA under different conditions of environmental factors, genetic background, and experimental design.

## 5. Conclusion

In summary, the HLA-DQB1*02, HLA-DRB1*03, DRB1*04, and DRB1*09 allele groups may predispose individuals to LADA; HLA-DQB1*06 and DQB1*05 have protective effects against the development of LADA; and HLA-DQB1*03, HLA-DQB1*04, HLA-DRB1*10, HLA-DRB1*11, HLA-DRB1*14, HLA-DRB1*15, and HLA-DRB1*16 may not play a role in this disease. However, DRB1*12 and DRB1*13 may play a protective role against LADA, and DRB1*01, DRB1*07, and DRB1*08 have uncertain associations with increased susceptibility to LADA. Further studies with larger sample sizes should be conducted to confirm these results.

## Figures and Tables

**Table 1:** Summary characteristics of the studies included in the meta-analysis.

First Author, Year	Country	Participants (*n*)		Subject: *DQB1* (*n*)		Subject: *DRB1* (*n*)		Age at Onset	Duration of Diabetes	HLA Genotyping	Allele Group	NOS
		LADA	Controls	LADA	Controls	LADA	Controls					
Yin N.N., 2017	China	652	1181	628	1159	652	1171	30–82	Not mentioned	PCR-SBT	DQB1*02, 03, 04, 06; DRB1*03, 04, 08, 09, 12	8
Cejkova P., 2008	Czech Republic	43	124	31	124	29	107	53 (35–71)	15.4 (4–32)	SSP-PCR	DQB1*02, 03, 04, 05, 06; DRB1*01, 03, 04, 07, 08, 09, 10, 11, 12, 13, 14, 15, 16	7
Katarina K., 2007	Czech Republic	31	153	27	99	27	99	47.0 (25–64)	15.0 (3–32)	SSP-PCR	DQB1*02, 03; DRB1*03, 04	6
Desai M., 2007	UK, Ireland	378	327	377	327	378	327			SSP-PCR	DQB1*02, 03, 04, 05, 06; DRB1*01, 03, 04, 07, 08, 09, 10, 11, 12, 13, 14, 15, 16	6
		UKPDS: *n* = 211						46.3 (25–65)	Newly diagnosed			
		W2: *n* = 130						47.4 (26–68)	8.9			
		YT2D: *n* = 37						38.4 (29–45)	11.9			
Li Q., 2005	China	39	60	39	60			>25	5.6	SSP-PCR	DQB1*02, 03, 06	6
Hosszufalusi N., 2003	Hungary	54	336	50	336			51.9 (39–61.8)	4.00 (1.0–9.5)	SSP-PCR	DQB1*03	6
Cerna M., 2003	Czech Republic	70	99	70	99	70	99	52 (35–71)	14 (4–29)	SSP-PCR	DQB1*02, 03, 04, 05, 06; DRB1*01, 03, 04, 07, 08, 09, 10, 11, 12, 13, 14, 15, 16	7
Vatay A., 2002	Hungary	42	336	41	336	41	336	>35	Not mentioned	SSP-PCR	DQB1*02, 03, 04, 05, 06; DRB1*01, 03, 04, 07, 08, 09, 10, 11, 12, 13, 14, 15, 16	7
Xiao J.Z., 1997	China	15	41	15	41			>20	Not mentioned	PCR/SSO	DQB1*02, 03, 04, 05, 06	6

LADA: latent autoimmune diabetes in adults; SSP: sequence-specific primer; PCR: polymerase chain reaction; SBT: sequence-based typing; SSO: sequence-specific oligonucleotide; NOS: Newcastle–Ottawa Scale; UKPDS: the UK Prospective Diabetes Study; W2: the Warren 2 Repository; YT2D: the Exeter Young-Onset Type 2 Diabetes Study.

**Table 2:** Meta-analysis of the association between *HLA-DQB1* polymorphisms and susceptibility to LADA.

Allele Group	Studies	Cases	Controls	OR	95% CI	*p*	*p_c_*	*p_h_*	*I²*	*p_power_*
DQB1*02	**8**	**1228**	**2245**	**1.685**	**1.298–2.187**	**<0.001**	**<0.005**	**0.006**	**64.3%**	**1.000**
DQB1*03	9	1278	2581	1.109	0.913–1.347	0.296	-	0.013	58.8%	0.231
DQB1*04	6	1162	2086	1.159	0.652–2.059	0.616	-	0.025	61.0%	0.136
DQB1*05	5	**534**	**927**	**0.764**	**0.609–0.958**	**0.020**	0.100	0.182	35.9%	0.260
DQB1*06	**7**	**1201**	**2146**	**0.604**	**0.438–0.833**	**0.002**	**0.010**	**0.006**	**66.7%**	**0.928**

OR: odds ratio; CI: confidence interval; *p:* probability for the overall effect test; *p_c_*: *P*-value corrected by the Bonferroni method; *p_h_*: probability for the heterogeneity test; *p_power_*: statistical power.

**Table 3:** Subgroup analysis by population origin for associations of *HLA-DQB1* polymorphisms with LADA risk.

Allele Group	Population Origin	OR	95% CI	*p*	*p_c_*	*p_h_*	*I²*
DQB1*02	Asian	**2.312**	**1.873–2.854**	**<0.001**	**<0.005**	**0.608**	**0.0%**
	European	1.387	0.987–1.950	0.059	0.295	0.014	68.2%
DQB1*03	Asian	1.049	0.706–1.560	0.811	-	0.156	46.2%
	European	1.200	0.888–1.620	0.235	-	0.010	66.8%
DQB1*04	Asian	1.198	0.369–3.888	0.764	-	0.040	76.3%
	European	0.892	0.539–1.475	0.656	-	0.452	0.0%
DQB1*05	Asian	0.286	0.015–5.463	0.406	-	-	-
	European	**0.769**	**0.612–0.964**	**0.023**	0.115	0.121	48.4%
DQB1*06	Asian	1.004	0.503–2.007	0.990	-	0.029	71.9%
	European	**0.430**	**0.346–0.535**	**<0.001**	**<0.005**	**0.708**	**0.0%**

**Table 4:** Relative Predispositional Effects (RPEs) of *HLA-DQB1* allele groups on LADA.

Allele Group	Round 1 of Comparison				Round 2: DQB1*02 Removed			Round 3: DQB1*06 Removed			Round 4: DQB1*05 Removed		
	Observed ^a^	Expected ^b^	χ²	*p* ^c^	Expected	χ²	*p*	Expected	χ²	*p*	Expected	χ²	*p*
DQB1*02	**583**	**440**	**46.475**	**<0.001**	-	-	-	-	-	-	-	-	-
DQB1*03	818	795	0.665	0.415	**724**	**12.204**	**<0.001**	770	2.992	0.084	836	0.388	0.534
DQB1*04	**129**	**105**	**5.486**	**0.019**	**96**	**11.344**	**<0.001**	**102**	**7.147**	**0.008**	111	2.919	0.088
DQB1*05	**266**	**352**	**21.011**	**<0.001**	**320**	**9.113**	**0.003**	**341**	**16.496**	**<0.001**	-	-	-
DQB1*06	**225**	**328**	**32.345**	**<0.001**	**298**	**17.883**	**<0.001**	-	-	-	-	-	-
Total	**2021**	**2021**	**105.982**	**<0.001**	**1438**	**50.543**	**<0.001**	**1213**	**26.635**	**<0.001**	947	3.306	0.069

^a^ Both observed allele groups and control allele groups were corrected by standardization. ^b^ Expected, based on observed frequencies for 3728 control alleles. ^c^ Two-sided probability values calculated using a χ-square test. Bold: statistically significant χ²- or *p*-value.

**Table 5:** Meta-analysis of the association between *HLA-DRB1* polymorphisms and susceptibility to LADA.

Allele Group	Studies	Cases	Controls	OR	95% CI	*p*	*p_c_*	*p_h_*	*I²*	*p_power_*
DRB1*01	4	518	869	0.801	0.607–1.055	0.114	-	0.284	21.0%	0.153
DRB1*03	**6**	**1197**	**2139**	**2.685**	**2.060–3.501**	**<0.001**	**<0.013**	**0.054**	**54.0%**	**1.000**
DRB1*04	**6**	**1197**	**2139**	**1.954**	**1.507–2.535**	**<0.001**	**<0.013**	**0.070**	**50.9%**	**1.000**
DRB1*07	4	518	869	0.534	0.272–1.047	0.068	0.884	0.003	78.9%	0.856
DRB1*08	5	1170	2040	1.121	0.556–2.261	0.749	-	0.001	77.9%	0.093
DRB1*09	**5**	**1170**	**2040**	**1.346**	**1.152–1.572**	**<0.001**	**<0.013**	**0.396**	**1.8%**	**0.723**
DRB1*10	4	518	869	1.052	0.334–3.316	0.931	-	0.539	0.0%	0.052
DRB1*11	4	518	869	0.727	0.349–1.515	0.395	-	0.001	82.4%	0.247
DRB1*12	**5**	**1170**	**2040**	**0.600**	**0.479–0.751**	**<0.001**	**<0.013**	**0.157**	**39.6%**	**0.660**
DRB1*13	**4**	**518**	**869**	**0.583**	**0.432–0.787**	**<0.001**	**<0.013**	**0.938**	**0.0%**	**0.494**
DRB1*14	4	518	869	0.607	0.328–1.124	0.112	-	0.565	0.0%	0.110
DRB1*15	4	518	869	0.482	0.215–1.082	0.077	-	0.001	82.6%	0.696
DRB1*16	4	518	869	0.663	0.375–1.172	0.157	-	0.627	0.0%	0.099

Bold: statistically significant χ²- or *p*-value.

**Table 6:** Subgroup analysis by population origin for associations of *HLA-DRB1* polymorphisms with LADA risk.

Allele Group	Population Origin	OR	95% CI	*p*	*p_c_*	*p_h_*	*I²*
DRB1*03	Asian	**2.095**	**1.636–2.682**	**<0.001**	**<0.013**	-	-
	European	**2.942**	**2.205–3.924**	**<0.001**	**<0.013**	**0.177**	**36.7%**
DRB1*04	Asian	**1.886**	**1.468–2.424**	**<0.001**	**<0.013-**	-	-
	European	**2.049**	**1.383–3.035**	**<0.001**	**<0.013**	**0.040**	**60.1%**
DRB1*08	Asian	**0.574**	**0.439–0.751**	**<0.001**	**<0.013**	-	-
	European	1.454	0.620–3.412	0.390	-	0.026	67.7%
DRB1*09	Asian	**1.359**	**1.160–1.593**	**<0.001**	**<0.013**	-	-
	European	1.050	0.474–2.328	0.904	-	0.297	18.6%
DRB1*12	Asian	**0.632**	**0.502–0.795**	**<0.001**	**<0.013**	-	-
	European	**0.215**	**0.077–0.598**	**0.003**	**0.039**	**0.539**	**0.0%**

**Table 7:** RPEs of *HLA-DRB1* allele groups on LADA.

Allele Group	Round 1 of Comparison				Round 2: DRB1*03 Removed			Round 3: DRB1*04 Removed			Round 4: DRB1*09 Removed			Round 5: DRB1*01 Removed			Round 6: DRB1*07 Removed			Round 7: DRB1*08 Removed		
	Observed ^a^	Expected ^b^	χ²	*p* ^c^	Expected	χ²	*p*	Expected	χ²	*p*	Expected	χ²	*p*	Expected	χ²	*p*	Expected	χ²	*p*	Expected	χ²	*p*
DRB1*01	179	179	0.000	1.000	164	1.372	0.242	**149**	**6.040**	**0.014**	**138**	**12.181**	**<0.001**	-	-	-	-	-	-	-	-	-
DRB1*03	**415**	**256**	**98.754**	**<0.001**	-	-	-	-	-	-	-	-	-	-	-	-	-	-	-	-	-	-
DRB1*04	**363**	**239**	**64.335**	**<0.001**	**219**	**94.685**	**<0.001**	-	-	-	-	-	-	-	-	-	-	-	-	-	-	-
DRB1*07	250	276	2.449	0.118	253	0.036	0.850	230	1.739	0.187	**212**	**6.811**	**0.009**	**203**	**10.882**	**0.001**	-	-	-	-	-	-
DRB1*08	100	116	2.207	0.137	107	0.458	0.499	97	0.093	0.761	89	1.360	0.244	86	2.279	0.131	**80**	**5.000**	**0.025**	-	-	-
DRB1*09	**301**	**251**	**9.960**	**0.002**	**230**	**21.917**	**<0.001**	**209**	**40.498**	**<0.001**	-	-	-	-	-	-	-	-	-	-	-	-
DRB1*10	6	12	3.000	0.083	11	2.273	0.132	10	1.600	0.206	9	1.000	0.317	9	1.000	0.317	8	0.500	0.480	8	0.500	0.480
DRB1*11	**166**	**239**	**22.297**	**<0.001**	**220**	**13.255**	**<0.001**	**199**	**5.472**	**0.019**	184	1.761	0.185	176	0.568	0.451	165	0.006	0.938	160	0.225	0.635
DRB1*12	**96**	**133**	**10.293**	**0.001**	**122**	**5.541**	**0.019**	111	2.027	0.155	102	0.353	0.553	98	0.041	0.840	92	0.174	0.677	89	0.551	0.458
DRB1*13	**137**	**187**	**13.369**	**<0.001**	**172**	**7.122**	**0.008**	156	2.314	0.128	144	0.340	0.560	138	0.007	0.932	129	0.496	0.481	125	1.152	0.283
DRB1*14	**27**	**44**	**6.568**	**0.010**	**40**	**4.225**	**0.040**	36	2.250	0.134	34	1.441	0.230	32	0.781	0.377	30	0.300	0.584	29	0.138	0.710
DRB1*15	**123**	**199**	**29.025**	**<0.001**	**182**	**19.126**	**<0.001**	**165**	**10.691**	**0.001**	**153**	**5.882**	**0.015**	146	3.623	0.057	137	1.431	0.232	133	0.752	0.386
DRB1*16	**31**	**64**	**17.016**	**<0.001**	**59**	**13.288**	**<0.001**	**53**	**9.132**	**0.003**	**49**	**6.612**	**0.010**	**47**	**5.447**	**0.020**	44	3.841	0.050	43	3.349	0.067
total	**2194**	**2194**	**279.273**	**<0.001**	**1779**	**183.298**	**<0.001**	**1416**	**81.856**	**<0.001**	**1115**	**37.742**	**<0.001**	**936**	**24.628**	**<0.001**	**686**	**11.748**	**<0.001**	586	6.666	0.001

^a^ Both observed allele groups and control allele groups were corrected by standardization. ^b^ Expected, based on observed frequencies for 3728 control allele groups. ^c^ Two-sided probability values calculated using a chi square test. Bold: statistically significant χ²- or *p*-value.

## Supplementary Materials

The following are available online at https://www.mdpi.com/2073-4425/10/9/710/s1: Table S1: Quality assessment scheme for the included literature (Newcastle–Ottawa Scale), Table S2: The main characteristics of LADA patients and controls, Table S3: Meta-regression with concomitant variables for the heterogeneity analysis, Table S4: A leave-one-out sensitivity analysis for *HLA-DQB1*05*, *HLA-DRB1*08*, and *HLA-DRB1*09*, Table S5: Test for publication bias in the association between the *DQB1* and *DRB1* allele groups and the risk of developing LADA, Table S6: The frequencies of *DQB1* and *DRB1* allele groups among populations originating from Asia and Europe.
