# Supplementary material for: HLA-DQB1 and HLA-DRB1 Variants Confer Susceptibility to Latent Autoimmune Diabetes in Adults: Relative Predispositional Effects among Allele Groups
Source: Genes (Basel). 2019 Sep 13;10(9):710. doi: 10.3390/genes10090710 (PMC6771152; doi:10.3390/genes10090710)
Supplement: Supplementary file 1 [file genes-10-00710-s001.pdf]

Supplementary Materials: HLA-DQB1 and HLA-DRB1 variants confer susceptibility to latent autoimmune diabetes in adults: relative predispositional effects among allele groups

Minting Zhang#, Shuhuang Lin#, Xiaolin Yuang, Ziqi Lin, Zunnan Huang*

Table S1. Quality assessment scheme for the included literature (Newcastle-Ottawa Scale).

Literature	Selection				Comparability	Exposure			Total
	I	II	III	IV	V	VI	VII	VIII	
Yin N.N. (2017)	*	*	*	*	*	*	*	*	********
Cejkova P. (2008)	*	*	*		*	*	*	*	*******
Katarina K. (2007)	*	*	*		*		*	*	******
Desai M. (2007)		*	*	*	*		*	*	******
Li Q. (2005)	*		*	*	*		*	*	******
Hosszufalusi N. (2003)	*		*	*	*		*	*	******
Cerna M. (2003)	*		*	*	*	*	*	*	*******
Vatay A. (2002)	*		*	*	*	*	*	*	*******
Xiao J.Z. (1997)	*		*	*	*		*	*	******

I: Adequacy of the case definition; II: Representativeness of the cases; III: Selection of controls; IV: Definition of controls; V: Comparability of cases and controls on the basis of the design or analysis; VI: Ascertainment of exposure; VII: Same method of ascertainment for cases and controls; VIII: Nonresponse rate.

Table S2. The main characteristics of LADA patients and controls.

First Author, Year	Male/Female		Mean Age (years)		BMI (kg/m²)		No Insulin Treatment after Diagnosis (months)	Mean C-peptide (pmol/L)	GADA (ng/mL)	IA-2A	ICAs
	LADA	Control	LADA	Control	LADA	Control					
Yin N.N., 2017	370/282	640/541	50.6±11.3	41.9±16.4			6		+ / −	+ / −	
Cejkova P., 2008					31.6 (26.6-45.4)		6	At onset: 542.2 (204.5–1522)	554.5(50.4–2000)		
Katarina K., 2007	17/14						6	478.0 (4.4–1522)	392.0 (5–2800)		
Desai M., 2007		150/177		55.3±19.8(20–91)		25.4±4.0					

	UKPDS	110/101		25.1±4.8	3		+		
	W2	74/56		28.6±5.3	12		+		
	YT2D	16/21		27.5±5.8	3		+/−	+/−	
Li Q., 2005		23/16	37.5		6		+		
Hosszufalusi N., 2003		25/29	59.0 (47.5–67.0)	23.5 (22.6-27.1)	6	At onset: 530.0 (240–1400) After onset, 1-10 years: 340.0 (210–1870) After onset, >10 years: 400.0 (160–1170)	+/−	+/−	+/−
Cerna M., 2003		30/40		27 (22–37)	6	609.0 (51–2800)	193.0 (3–3000)	+/−	
Vatay A., 2002		20/22	56.9±2.3		6		+/−		+/−
Xiao J.Z., 1997					6		+/−	+/−	

BMI: body mass index; GADA: antiglutamic acid decarboxylase antibody; IA-2A: islet antigen 2A; ICAs: antibodies against islet cells; +: positive reaction; −: negative reaction; +/−: some patients were positive for GADA, IA-2A or ICAs.

Table S3. Meta-regression with concomitant variables for the heterogeneity analysis.

**Allele Group**	***N***	**Publication Year**		**Ethnicity**		**Sample Size**		**NOS Score**	
		β	***p***	***β***	***p***	***β***	***p***	***β***	***p***
*DQB1*02*	8	1.019	0.489	1.781	0.079	1.000	0.193	0.964	0.871
*DQB1*03*	9	0.996	0.887	0.880	0.724	1.000	0.580	0.780	0.244
*DQB1*04*	6	1.063	0.052	1.304	0.659	1.001	0.090	1.689	**0.028**
*DQB1*05*	5	1.013	0.878	0.345	0.539	0.999	0.478	1.570	0.253
*DQB1*06*	7	0.974	0.509	1.872	0.075	1.000	0.675	0.830	0.551
*DRB1*03*	6	0.985	0.617	0.712	0.299	1.000	0.234	0.814	0.271
*DRB1*04*	6	0.981	0.543	0.922	0.849	1.000	0.709	1.014	0.953
*DRB1*07*	4	1.064	0.697	0.538	0.139	1.002	0.069	0.447	0.250
*DRB1*08*	5	0.919	0.181	0.398	0.313	0.999	0.136	0.922	0.902
*DRB1*11*	4	1.079	0.717	0.727	0.468	0.998	0.288	1.783	0.615
*DRB1*12*	5	1.108	0.120	2.917	0.139	1.001	0.132	1.732	0.279
*DRB1*15*	4	0.882	0.471	0.477	0.130	0.998	0.055	3.595	0.055

Note: β, regression coefficient in the form of an index; *p*, the *p* value of the meta-regression; Bold: statistically significant *p* value.

Table S4. Leave-one-out sensitivity analysis for *HLA-DQB1*05*, *HLA-DRB1*08* and *HLA-DRB1*09*.

**Allele Group**	**Leave-one-out study**	**OR**	**95% CI**	***p***	***I²***	p_h
*DQB1*05*						
	Cejkova P. 2008	**0.719**	**0.567-0.913**	**0.007**	19.60%	0.292
	Desai M. 2007	0.919	0.627-1.256	0.594	12.90%	0.328
	Cerna M. 2003	0.883	0.552-1.410	0.601	50.90%	0.107
	Vatay A. 2002	**0.706**	**0.551-0.904**	**0.006**	22.40%	0.276
	Xiao J.Z. 1997	**0.769**	**0.612-0.964**	**0.023**	48.40%	0.121
*DRB1*08*						
	Yin N.N. 2017	1.454	0.620-3.412	0.390	67.70%	0.026
	Cejkova P. 2008	1.038	0.484-2.225	0.925	81.50%	0.001
	Desai M. 2007	1.401	0.511-3.839	0.512	83.30%	0.000
	Cerna M. 2003	**0.623**	**0.490-0.792**	**0.000**	37.00%	0.190
	Vatay A. 2002	1.065	0.485-2.340	0.875	81.90%	0.001
*DRB1*09*						
	Yin N.N. 2017	1.050	0.474-2.328	0.904	18.60%	0.297
	Cejkova P. 2008	**1.344**	**1.150-1.571**	**0.000**	25.10%	0.261
	Desai M. 2007	**1.372**	**1.173-1.606**	**0.000**	0.00%	0.702
	Cerna M. 2003	**1.334**	**1.141-1.559**	**0.000**	0.00%	0.444
	Vatay A. 2002	**1.346**	**1.152-1.573**	**0.000**	26.20%	0.255

Note: OR, odds ratio; CI, confidence interval; *p*, probability for overall effect test; p_h , probability for heterogeneity test; Bold: statistically significant *p* value.

Table S5. Test for publication bias in the association between the *DQB1* and *DRB1* allele groups and the risk of developing LADA.

Allele Group	*n*	*p-Begg*	*p-Egger*
*DQB1*02*	8	0.902	0.382
*DQB1*03*	9	0.175	0.229
*DQB1*04*	6	0.707	0.281
*DQB1*05*	5	0.462	0.642
*DQB1*06*	7	0.368	0.322
*DRB1*03*	6	0.707	0.560
*DRB1*04*	6	1.000	0.321
*DRB1*08*	5	0.221	0.127
*DRB1*09*	5	0.806	0.947

Note: *n*, the number of studies for the corresponding allele group; *p-Begg*, the *p* value of Begg's test; *p-Egger*, the *p* value of Egger's test.

Table S6. The frequencies of *DQB1* and *DRB1* allele groups among populations originating from Asia and Europe.

Allele	Asian		European	
	Count (2*n*)	Frequency	Count (2*n*)	Frequency
*DQB1*02*	393	0.101	973	0.318
*DQB1*03*	1566	0.403	1189	0.310
*DQB1*04*	239	0.065	68	0.024
*DQB1*05*	4	0.036	459	0.163
*DQB1*06*	471	0.121	522	0.186
*DRB1*01*			265	0.096
*DRB1*03*	275	0.075	614	0.203
*DRB1*04*	266	0.073	567	0.187
*DRB1*07*			408	0.147
*DRB1*08*	304	0.083	95	0.034
*DRB1*09*	825	0.226	28	0.010
*DRB1*10*			18	0.006
*DRB1*11*			355	0.128
*DRB1*12*	408	0.112	51	0.018
*DRB1*13*			278	0.100
*DRB1*14*			65	0.023
*DRB1*15*			295	0.106
*DRB1*16*			95	0.034
